# Tripartite motif 13 orchestrates endoplasmic reticulum-associated degradation and endoplasmic reticulum-phagy to modulate dendritic cell-mediated immune responses in sepsis

**DOI:** 10.1093/burnst/tkaf077

**Published:** 2025-12-08

**Authors:** Sen Tong, Tuo Zhang, Ning Chen, Jing-peng Liu, Shu-ting Wei, Tian-zhen Hua, Yu Duan, Bing Sun, Ning Dong, Yao Wu, Xiao-mei Zhu, Yong-ming Yao

**Affiliations:** Chinese PLA Medical School and Chinese PLA General Hospital, 28 Fuxing Road, Haidian District, Beijing 100853, China; Medical Innovation Research Department of the Chinese PLA General Hospital, 28 Fuxing Road, Haidian District, Beijing 100853, China; Chinese PLA Medical School and Chinese PLA General Hospital, 28 Fuxing Road, Haidian District, Beijing 100853, China; Medical Innovation Research Department of the Chinese PLA General Hospital, 28 Fuxing Road, Haidian District, Beijing 100853, China; Medical Innovation Research Department of the Chinese PLA General Hospital, 28 Fuxing Road, Haidian District, Beijing 100853, China; Department of Traditional Chinese Medical Science, Sixth Medical Center of the Chinese PLA General Hospital, 6 Fucheng Road, Haidian District, Beijing 100048, China; Chinese PLA Medical School and Chinese PLA General Hospital, 28 Fuxing Road, Haidian District, Beijing 100853, China; Medical Innovation Research Department of the Chinese PLA General Hospital, 28 Fuxing Road, Haidian District, Beijing 100853, China; Chinese PLA Medical School and Chinese PLA General Hospital, 28 Fuxing Road, Haidian District, Beijing 100853, China; Medical Innovation Research Department of the Chinese PLA General Hospital, 28 Fuxing Road, Haidian District, Beijing 100853, China; Medical Innovation Research Department of the Chinese PLA General Hospital, 28 Fuxing Road, Haidian District, Beijing 100853, China; Medical Innovation Research Department of the Chinese PLA General Hospital, 28 Fuxing Road, Haidian District, Beijing 100853, China; Burn and Wound Repair Department, Fujian Medical University Union Hospital, 29 Xinquan Road, Gulou District, Fuzhou 350001, Fujian, China; Medical Innovation Research Department of the Chinese PLA General Hospital, 28 Fuxing Road, Haidian District, Beijing 100853, China; Medical Innovation Research Department of the Chinese PLA General Hospital, 28 Fuxing Road, Haidian District, Beijing 100853, China; Chinese PLA Medical School and Chinese PLA General Hospital, 28 Fuxing Road, Haidian District, Beijing 100853, China; Medical Innovation Research Department of the Chinese PLA General Hospital, 28 Fuxing Road, Haidian District, Beijing 100853, China; Chinese PLA Medical School and Chinese PLA General Hospital, 28 Fuxing Road, Haidian District, Beijing 100853, China; Medical Innovation Research Department of the Chinese PLA General Hospital, 28 Fuxing Road, Haidian District, Beijing 100853, China; National Clinical Research Center for Geriatric Diseases, The Chinese PLA General Hospital, 28 Fuxing Road, Haidian District, Beijing 100853, China

**Keywords:** Sepsis, Dendritic cells, Tripartite motif 13, Endoplasmic reticulum-associated degradation, Endoplasmic reticulum-selective autophagy, Pyroptosis, Stimulator of interferon genes

## Abstract

**Background:**

Sepsis is a life-threatening condition characterized by profound immune dysregulation and organ dysfunction. The functional impairment of dendritic cells (DCs) in septic patients is well-documented and contributes significantly to sepsis-induced immunosuppression; yet the underlying mechanisms remain poorly understood. Tripartite motif 13 (TRIM13) has been identified as an immune regulator with predominantly suppressive effects. Here, we aimed to investigate the potential role of TRIM13 restriction in promoting the DC-mediated immune response during sepsis.

**Methods:**

Splenic DCs were isolated from wild-type (WT) and DC-specific *Trim13* conditional knockout (*Trim13* cKO) mice post-cecum ligation and puncture (CLP). These cells were subsequently analyzed by proteomics, immunoblotting, flow cytometry, and transmission electron microscopy (TEM). DC2.4 cells were infected with either *Trim13* shRNA or a *Trim13* overexpression lentiviral vector and treated with different pharmacological inhibitors. Protein interactions were examined *via* coimmunoprecipitation (Co-IP) and confocal microscopy. Cytokine levels were measured by enzyme-linked immunosorbent assay (ELISA), and organ lesions were assessed through hematoxylin and eosin (H&E) staining, immunohistochemistry (IHC) for CD45, and TUNEL assays.

**Results:**

TRIM13 expression was rapidly upregulated in DCs following septic challenge. Deletion of TRIM13 in DCs disrupted the endoplasmic reticulum (ER)-associated degradation (ERAD) and ER-selective autophagy (ER-phagy)-mediated degradation of the stimulator of interferon genes (STING), leading to sustained STING activation and enhanced DC function. STING signaling promoted the p-IRF3 nuclear translocation, NLRP3 inflammasome priming, and transient DC pyroptosis, thereby exacerbating hyperinflammation in the acute phase of sepsis. Over the longer term, prolonged STING signaling inhibited DCs from adopting the immunosuppressive phenotype and promoting the DC-mediated immune response. Ultimately, TRIM13 deficiency in DCs ameliorated sepsis-induced immunosuppression, preserved organ function in the late phase of sepsis, and reduced overall mortality in septic mice.

**Conclusions:**

TRIM13 acts as a key negative regulator of DC function during sepsis. Restricting TRIM13 sustains DC immunostimulatory property, counteracts sepsis-induced immunosuppression, and improves survival outcomes. These findings highlight TRIM13 as a potential therapeutic target for sepsis management.

## Graphical Abstract

**Graphical Abstract:**
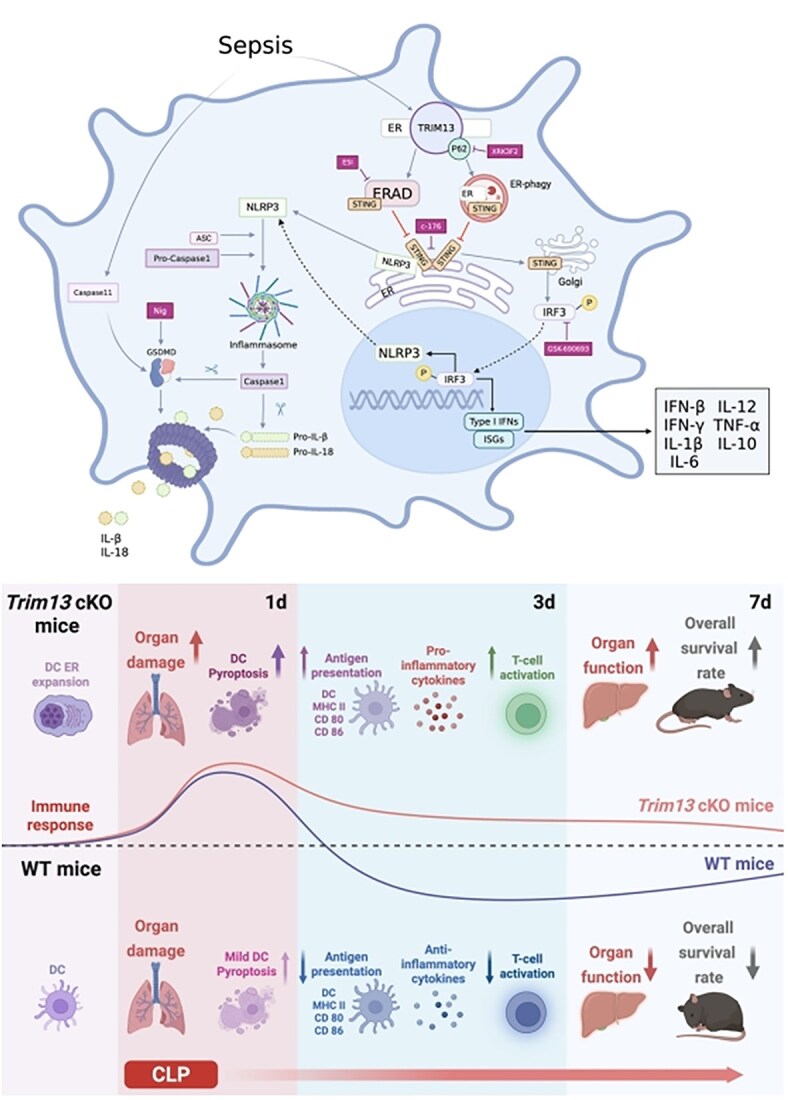

## Highlights

- DC TRIM13 deficiency enhances effector T cell activation and proliferation, increases pro-inflammatory cytokine production and lymphocyte counts, thereby improving organ function and ultimately promoting overall survival of septic mice.
- TRIM13 deficiency primarily disrupts ERAD-mediated degradation of STING, leading to sustained STING activation in *Trim13^Cd11c^* DCs.
- TRIM13-mediated ER-phagy serves as a compensatory pathway in STING degradation when ERAD is impaired.
- Sustained STING signaling in *Trim13^Cd11c^* DCs promotes early DC pyroptosis and prevents DCs from transitioning into an immunosuppressive phenotype.

## Background

Sepsis, described as ‘the quintessential medical disorder of the 21st century’, has a rich historical context that spans centuries [1]. First made public in 1991, the modern definition of sepsis has undergone substantial evolution. The current consensus defines sepsis as life-threatening organ dysfunction due to a dysregulated host response to infection [2]. Advances in early identification, timely antibiotic therapy, and supportive care have led to notable decreases in the incidence and mortality of sepsis. However, with approximately 48.9 million new cases and 11 million deaths recorded annually, sepsis remains the leading cause of death worldwide [3].

The pathophysiology of sepsis involves a complex interaction between hyperinflammation and immunosuppression [4]. Initial efforts have predominantly concentrated on mitigating hyperinflammatory responses; however, clinical trials targeting the elimination of proinflammatory cytokines have failed to yield improved outcomes. This inefficacy can be attributed to the intricacies and dynamic nature of sepsis, where an early hyperinflammatory phase is often accompanied by an undetected compensatory anti-inflammatory response [5]. Thus, the initial proinflammatory response can rapidly shift into a prolonged state of immune depression, increasing the risk of intractable secondary infections and exacerbating the severity of complications, thereby contributing to overall high mortality [6–8].

A promising countermeasure for sepsis-induced immunosuppression is preserving the functionality of dendritic cells (DCs). Serving as professional antigen-presenting cells (APCs), DCs play a pivotal role in bridging innate and adaptive immunity by activating and inducing the clonal expansion of naïve T cells [4]. However, in sepsis, DCs often shift toward an immunosuppressive phenotype, characterized by reduced major histocompatibility complex (MHC) class II expression and pro-inflammatory cytokine production, thereby weakening host defenses against subsequent bacterial challenge [9, 10]. Notably, while DC counts may recover weeks after sepsis, the functional capacity remains compromised for an extended period, leading to adverse clinical outcomes [6]. Despite these findings, the mechanisms underlying DC dysfunction in sepsis remain poorly understood, and effective therapeutic approaches targeting host immune suppression remain limited in clinical practice.

Tripartite motif 13 (TRIM13), the sole endoplasmic reticulum (ER)-residing member of the TRIM family, has emerged as a promising candidate for enhancing DC functionality. Originally identified as a tumor suppressor in leukemia and cancer, TRIM13 plays a unique role in promoting ER quality control (ERQC) [including ER-phagy and ER-associated degradation (ERAD)] [11–14]. TRIM13 also acts as a negative regulator of the stimulator of interferon genes (STING) pathway in macrophages during viral infections [13]. This role is particularly relevant, as STING signaling has been implicated in DC maturation and pro-inflammatory functional state, which is increasingly recognized as a powerful strategy to enhance DC-mediated antitumor immunity [15–17].

In light of these findings, we speculated that TRIM13 plays a critical role in the host immune response during sepsis, particularly in aberrant DC STING signaling and DC dysfunction. To test this hypothesis, we employed a DC-specific conditional knockout mouse model (*Trim13*^flox/flox^; *Cd11c*-Cre, hereafter referred to as ‘*Trim13* cKO’) to investigate how TRIM13 influences ERQC in DCs and impacts DC STING signaling, pyroptosis, and function. We further evaluated the therapeutic potential of targeting DC STING signaling *via* TRIM13 regulation as an interventional measure for sepsis treatment. Our findings might offer novel therapeutic strategies aimed at enhancing immune responses and improving prognosis in septic patients.

## Methods

### Mice

*Trim13* cKO mice on the C57BL/6 J background were generated by Cyagen Biosciences Inc., Suzhou, Jiangsu, China. The *Trim13* gRNA target sequences were as follows: gRNA1 (reverse strand): TGCTGAAACAGGGTTAGGACTGG; gRNA2 (forward strand): GTACACTTAATGTGCATAAGTGG; gRNA3 (reverse strand): GCTGAAACAGGGTTAGGACTGGG; and gRNA4 (forward strand): TGTGCATAAGTGGCACATGTGGG. The efficiency of *Trim13* knockout was examined *via* PCR and DNA agarose gel electrophoresis. The sequences of primers were as follows: Loxp site upstream primer (F1): 5′-ACAGTGAGACTCTTTGTTTCTGAC-3′; Loxp site downstream primer (R1): 5′-TGGATGCTAAGGCACATTTAACTC-3′. Cd11c-Cre-F: 5′-ACTTGGCAGCTGTCTCCAAG-3′; Cd11c-Cre-R: 5′-GCGAACATCTTCAGGTTCTG-3′. Homozygous *Trim13* cKO mice showed a single band at 257 bp. Heterozygous mice showed two bands at 257 bp and 194 bp. This study was performed according to the Guide for the Care and Use of Laboratory Animals of the National Institutes of Health. All animal procedures were approved by the Scientific Investigation Board of the Chinese PLA General Hospital, Beijing, China (No. 2022-X18-94).

### Isolation of mouse splenic DCs

To isolate CD11c^+^ cells using the magnetic bead system (Cat No. 130-125-835; Miltenyi, Germany), mouse spleens were harvested and kept in cold PBS. A single-cell suspension was prepared and centrifuged at 300 × g for 10 min. The cell pellet was subsequently resuspended and counted using an automated cell counter (Count Star, China). The cell suspension was labeled with CD11c microbeads by adding 10 μl of bead mixture and 40 μl of buffer per 10^7^ cells, mixing well, and incubating for 15 min at 4°C. For magnetic separation, a MACS column was prepared, rinsed, and placed in the MACS Separator. The cell suspension was passed through the column. After removing the column from the separator, additional buffer was added, and the magnetically labeled CD11c^+^ cells were eluted from the column using a plunger. The enriched CD11c^+^ cells were collected and counted.

### Isolation of mouse splenic CD4^+^ T cells

To isolate splenic CD4^+^ T cells, mouse spleens were harvested and processed into single-cell suspensions. After density gradient centrifugation (TBD, China), the separated cells were carefully extracted, washed, and counted. The cells were then resuspended in 40 μl of buffer, with 10 μl of CD4^+^ T cell biotin-antibody cocktail (Cat No. 130-104-454) added per 10^7^ cells. After incubation for 5 min at 4°C, 30 μl of buffer and 20 μl of antibiotin microbeads per 10^7^ cells were added, followed by mixing and incubation for 15 min at 4°C. The suspension was passed through a MACS column in a MACS separator, and the enriched CD4^+^ T cells were collected after being rinsed with buffer.

### Cell culture and stimulation

Cells, including DC2.4 cells (dendritic cell 2.4, RRID: CVCL_J409) and primary mouse splenic DCs, were routinely cultured in RPMI-1640 medium supplemented with 10% FBS, 100 U/ml penicillin, and 100 μl/ml streptomycin (Gibco, USA) at 37°C and 5% CO_2_ in a humidified incubator. DC2.4 cells were stabilized for 24 h, while mouse splenic DCs were stabilized for 6 h before stimulation. For sepsis and pyroptosis stimulation, the cells were primed with lipopolysaccharide (LPS) (1 μg/ml) for the indicated intervals, followed by stimulation with nigericin (Nig) (20 μm) for 30 min. For ERAD blockade, primed DC2.4 cells were treated with eeyarestatin I (ESI) (20 μm) for 8 h, and mouse splenic DCs were treated with ESI (10 μm) for 8 h. To block TRIM13-induced ER-phagy, primed cells were treated with XRK3F2 (10 μm). To inhibit STING, primed DC2.4 cells and mouse splenic DCs were pretreated with C-176 (50 and 10 μm, respectively) for 30 min before the indicated time points. To block p-IRF-3, primed DC2.4 cells and mouse splenic DCs were treated with GSK-690693 (20 and 10 μm, respectively). To inhibit proteasomal degradation and stabilize ubiquitinated proteins, DC2.4 cells were treated with MG-132 (10 μm) for 12 h prior to the indicated intervals.

### Reagents

CD11c^+^ (Cat No. 130-125-835) and CD4^+^ (Cat No. 130-104-454) microbeads were purchased from Miltenyi (Bergisch-Gladbach, Germany). The CD11c^+^ Positive Selection Kit II (Cat No. 18780) was purchased from STEMCELL Technologies. Both cycloheximide (CHX) (Cat No. 66-81-9) and LPS (*Escherichia coli* O127:B8, L4516) were purchased from Sigma-Aldrich (Missouri, USA). Syvn1 (HRD1) mouse pre-designed siRNA set A (HY-RS18845), Nig (HY-100381), MG-132 (HY-13259), C-176 (HY-112906), XRK3F2 (HY-112904), and GSK-690693 (HY-10249) were purchased from MCE (New Jersey, USA). ESI (Cat No. 3922) and ELISA kits were purchased from R&D (Minneapolis, MN). Caspase-11 (ab180673), GSDMD (ab209845), NLRP3 (ab263899), pro-caspase-1 + p10 + p12 (ab179515), SQSTM1/p62 (ab109012), and TRIM13 N-terminal (ab194477) antibodies were purchased from Abcam (Cambridge, MA). The CFSE (Cat No. 4238) Cell Division Tracker Kit and FITC I-A/I-E (Cat No. 107606), PE CD80 (Cat No. 1047088), and PE/Cyanine7 CD86 (Cat No. 105014) antibodies were purchased from BioLegend (San Diego, CA). ASC/TMSA (D2W8U), IL-1β (3A6), NLRP3 (D4D8T), CD45 (D3F8Q), LC3A/B (D3U4C) Alexa Fluor®488 Conjugate, LC3B (D11), p-IRF-3 (Ser396) (D6O1M), p-STING (Ser365) (D8F4W), p-TBK1/NAK (Ser172) (D52C2), Sec61B (D5Q1W), TBK1 (D1B4), and IRF-3 (D83B9) antibodies were purchased from Cell Signaling Technology (CST, Danvers, MA). ER-Tracker Red was purchased from Invitrogen (California, CA). TRIM13/Rfp2 (sc-398 129), ubiquitin (sc-8017), IgG (Alexa Fluor 488-conjugated), and IgG (Alexa Fluor 594-conjugated) were purchased from Santa Cruz Biotechnology (Santa Cruz, CA). HRD1/SYVN1 (13473–1-AP) and SEL1L (29801-1-AP) antibodies were purchased from Proteintech (Wuhan, China). The protein A/G beads and Co-IP (IK-1004) kit were purchased from Biolinkedin (Shanghai, China). Ribonucleic acid (RNA) Isolation Kit V2 (RC112-01) and (8%, 15%) PAGE gel kits (E302, E305) were purchased from Vazyme (Nanjing, China).

### CLP–induced sepsis model

WT and *Trim13* cKO mice were anesthetized *via* the intraperitoneal injection of sodium pentobarbital (0.3%, 60 mg/kg). Laparotomy was performed after 10–15 min of anesthesia. The cecum was isolated and ligated to 60% of its length, followed by transintestinal perforation with a 22-gage needle. Gentle pressure was applied to guide the fecal contents into the peritoneal cavity. After cecal repositioning, the abdomen was closed in layers with 6–0 sutures. Postoperative fluid resuscitation included the administration of 1 ml of normal saline subcutaneously.

### Flow cytometric analysis

Flow cytometric analysis was employed to evaluate DC functionality by quantifying the expression of surface markers, including CD80, CD86, and histocompatibility complex class II (MHC II). Splenic DCs were isolated from the cecal ligation and puncture (CLP) model mice and prepared as single-cell suspensions. The cells were washed, counted, and aliquoted into separate flow cytometry tubes at 2 × 10^5^ cells in 200 μl of PBS. Fluorescently conjugated antibodies (1 μg/tube) targeting these markers were incubated with the cells for 30 min (room temperature, RT) and then analyzed. The fluorescence intensity corresponding to CD80 and CD86 indicated the costimulatory potential of DCs, which is essential for effective T cell activation. Concurrently, MHC II expression levels provide information on the antigen-presenting ability of the cells.

The pyroptosis rates of DCs were assessed by determining caspase-1 activation and membrane integrity. After CLP, DCs were isolated and incubated with 10 μl of 30 × FAM-FLICA-CASP-1 solution in 290 μl of PBS for 60 min (37°C, dark). Following incubation, the cells were washed with apoptosis wash buffer, centrifuged at 1500 rpm for 5 min, and then incubated with 5 μl of 7-AAD in 100 μl of binding buffer for 15 min at RT in the dark. After incubation, the cells were diluted with binding buffer and analyzed. FAM fluorescence indicated caspase-1 activation, whereas 7-AAD uptake reflected membrane integrity, collectively indicating pyroptotic activity.

### Western blotting

The cells were collected, washed, and lysed in RIPA lysis buffer (Huaxingbio, HX1862–1, Beijing, China) supplemented with protease and phosphatase inhibitor cocktails (Huaxingbio, HX1863 and HX1863, Beijing, China). After 30 min of incubation on ice, the lysates were centrifuged at 12000 rpm for 30 min at 4°C and denatured with 1 × SDS for 5 min. The protein samples were loaded onto and separated by 8%–15% (Cat No. E303-C1 and E305-C1) SDS polyacrylamide gel electrophoresis (Vazyme Biotechnology Co., Nanjing, China). The separated proteins were transferred to PVDF membranes and blocked with 10% milk or 5% BSA for 1 h. The membranes were incubated overnight at 4°C with the indicated primary antibodies, followed by incubation with the recommended secondary antibodies. Immunoreactivity was visualized with an ECL reagent and analyzed using a detection system (Amersham Biosciences, Uppsala, Sweden).

### Co-IP assay

Harvested cells were washed and lysed under nondenaturing conditions with a pre-prepared lysis solution (Biolinkedin, IK-1004, Shanghai, China). The lysates were subjected to three freeze–thaw cycles after 1 h of incubation on ice. The supernatants were collected and centrifuged at 12 000 × g for 30 min at 4°C. Primary antibodies were added to the supernatants designated for the IP group, while control IgG antibodies were used for the IgG control group. The mixtures were incubated overnight at 4°C before protein A magnetic beads were added and then incubated at 4°C with gentle continuous rotation. The beads were separated using a magnetic rack, and the immunoprecipitated proteins were eluted by boiling before being subjected to Western blot analysis.

### Laser scanning confocal microscopy

The cells were washed, fixed with 4% paraformaldehyde, and permeabilized with 0.5% Triton X-100 for 1 min. After permeabilization, the cells were blocked with 5% BSA for 30 min and incubated overnight at 4°C with primary antibodies. After being washed, the cells were incubated with secondary antibodies. LysoTracker Deep Red (1:1000; Invitrogen, L12492, Carlsbad, CA) and ER-Tracker Red (1:1000; Invitrogen, E34250, Carlsbad, CA) probes were applied to the cells for 1 h at 37°C in an incubator. The cells were subsequently washed and stained with DAPI (1:300, Abcam, ab104139, Cambridge, MA). The cells were then washed, mounted on slides, and observed using a laser scanning confocal microscope (LSCM, Leica, Mannheim, Germany).

### TEM

After centrifugation to remove the supernatant, DCs were initially fixed with 2.5% glutaraldehyde at 4°C and postfixed with 1% osmium tetroxide (OsO4). The cells were then dehydrated using a graded series of acetone, followed by infiltration with Epon resin for embedding. Semithin sections were obtained for optical positioning, and ultrathin sections were cut for detailed examination. The sections were stained sequentially with 0.3% lead citrate and 2% uranyl acetate to enhance contrast. The morphology of DCs and their organelles, including the structure of the ER and ER-phagy, was examined using an H-7800 TEM (Hitachi, Tokyo, Japan).

### Elisa

Blood was collected from mice *via* the retro-orbital sinus under anesthesia with 0.3% sodium pentobarbital administered intraperitoneally. The collected blood was allowed to clot, and the serum was separated by centrifugation at 3000 × g for 15 min. Primary DCs were isolated from mouse spleens using the Miltenyi Biotec magnetic bead system (Cat No. 130-125-835; Miltenyi, Germany). The isolated DCs were cultured and stimulated as specified. After collection, the concentrations of cytokines in both the serum and culture supernatants were quantified using ELISA kits obtained from R&D Systems following the manufacturers’ protocols.

### Peripheral blood collection and analysis

Blood was collected from mice *via* the retro-orbital sinus under anesthesia with 0.3% sodium pentobarbital administered intraperitoneally. Approximately 300 μl of blood was obtained and transferred into EDTA-coated tubes, followed by gentle mixing to ensure proper anticoagulation. The samples were then subjected to immediate analysis using an automated hematology analyzer (BC-5000 Vet, Mindray, China) according to the manufacturer’s instructions. Hematologic parameters assessed included white blood cell counts (WBC), lymphocyte counts (LYM), and platelet counts (PLT).

### Histological examination

Dissected lung, liver and intestine tissues were fixed in 10% neutral buffered formalin (NBF) overnight and subsequently embedded in paraffin blocks. (i) Tissue sections were deparaffinized, and some of the samples were stained with HE. (ii) Some deparaffinized sections were rehydrated and permeabilized with Triton X-100, and the TdT enzyme, along with a fluorescently labeled dUTP, was applied. After the TUNEL reaction, the sections were washed, and nuclei were stained with DAPI. (iii) The other paraffin-embedded sections were subjected to IHC for CD45 to identify and differentiate leukocytes in the tissue sections. In brief, antigen retrieval was performed, and the sections were incubated with blocking solution, followed by incubation with a primary anti-CD45 antibody (D3F8Q; CST, Danvers, MA). The sections were washed and incubated with a secondary antibody. Thereafter, the sections were counterstained with hematoxylin, dehydrated, cleared, mounted, and examined. Histological features were observed and analyzed using a microscope (DS-Fi2, Nikon Instruments Co., Japan). Two experienced histologists were tasked with evaluating tissue sections at varying magnifications. Four random fields were selected per slide to assess tissue pathology and assign injury scores. Histopathological scoring for the lung, intestine, and liver was performed on the basis of the systems established by Mikawa, Chiu, and Eckhoff, respectively [18–22]. For the quantitative analysis of IHC (CD45) or immunofluorescence with terminal deoxynucleotidyl transferase dUTP nick-end labeling (IF/TUNEL) staining, image analysis was performed using ImageJ software. CD45^+^ cells (IHC) or apoptotic cells (identified by bright fluorescent spots in TUNEL IF staining) were delineated. The area occupied by positively stained cells was determined as a percentage of the total tissue area (percentage of positive area).

### RNA extraction and qPCR analysis

Total RNA was extracted from the cells using an RNA isolation kit (RC112-01, Vazyme, Nanjing, China) according to the manufacturer’s instructions. After reverse transcription of RNA into cDNA, qPCR was performed with SYBR Green master mix on a Quant Studio 5 system (Applied Biosystems, Waltham, MA). The sequences of primers were as follows: *Trim13* (5′-GGTAGTTCTGCTGGGTCTCCTC-3′ and 5′-TTCCAAGTTGCCAATTCATCAAGTG-3′); *NLRP3* (5′-TTTATTTGTACCCAAGGCTGCT-3′ and 5′-GCTTAGGTCCACACAGAAAG-3′); and *GAPDH* (5′-AGGTCGGTGTGAACGGATTTG-3′ and 5′-TGTAGACCATGTAGTTGAGGTCA-3′). The relative mRNA expression levels of *Trim13* and *NLRP3* were calculated using the 2 − ΔΔCt method relative to those in the control group.

### Lentivirus and siRNA transduction

DC2.4 cells were engineered for both the overexpression and knockdown of *Trim13* using lentiviral vector infection. The *Trim13* overexpression (hereafter referred to as *Trim13* OE) and *Trim13* knockdown (hereafter referred to as *Trim13* KD) lentiviral vectors were designed and constructed by Genechem (Shanghai, China). Empty lentiviral vectors were used as a negative control (hereafter referred to as Con). The specific shRNA sequence used to target *Trim13* was 5′-gcAGGTCAATTACTCCCTAAA-3′. The recombinant lentiviruses were transduced into DC2.4 cells in accordance with the manufacturer’s instructions. Following transduction, the cells were selected and expanded in the presence of puromycin (1 mg/ml) for one week. The efficacy of *Trim13* overexpression or knockdown was confirmed through qPCR and Western blot analysis.

For *Hrd1* knockdown in *Trim13* OE DC2.4 cells, cells were seeded in 6-well plates and transfected 24 h later with pre-designed siRNAs (HRD1 mouse pre-designed siRNA set A, MCE, USA), following the manufacturer’s protocol. Transfection efficiency was verified by western blotting 48 h post-transfection, after which cells were harvested for subsequent Co-IP assays.

### Proteomic sample preparation and LC–MS/MS analysis

Proteins were reduced with 5 mM dithiothreitol (56°C, 30 min), alkylated with 11 mM iodoacetamide (15 min, dark), and digested sequentially with trypsin after dilution with 200 mM TEAB to reduce urea to <2 M. Peptides were desalted (Strata X SPE column), separated on a reversed-phase column (25 cm × 100 μm i.d.) *via* a 15-min acetonitrile/0.1% formic acid gradient (6–80% B, 500 nL/min), and analyzed using a timsTOF Pro 2 mass spectrometer in dia-PASEF mode (MS: 300–1500 m/z; MS/MS: 400–850 m/z). Data were processed with DIA-NN against a decoy-enriched *Mus musculus* database (17 191 entries), with carbamidomethylation (fixed), N-terminal Met excision, and 1% FDR. Proteins with a fold change >1.5 or < 0.67 and a *P*-value <0.05 were considered differential proteins.

### Statistical analysis

All the results were obtained from at least three independent experiments. Comparisons between two groups were conducted using Student’s *t*-test. Differences among multiple groups were evaluated by one-way analysis of variance (ANOVA) followed by Tukey’s post-hoc test. When two independent variables were involved, two-way ANOVA with appropriate post-hoc multiple-comparison procedures was applied. The survival rate of septic mice was analyzed using Kaplan–Meier survival curves. A *P*-value <0.05 was considered statistically significant.

## Results

### Trim13 deficiency in DCs significantly alters the inflammatory response and outcomes of septic mice

**Figure 1:**
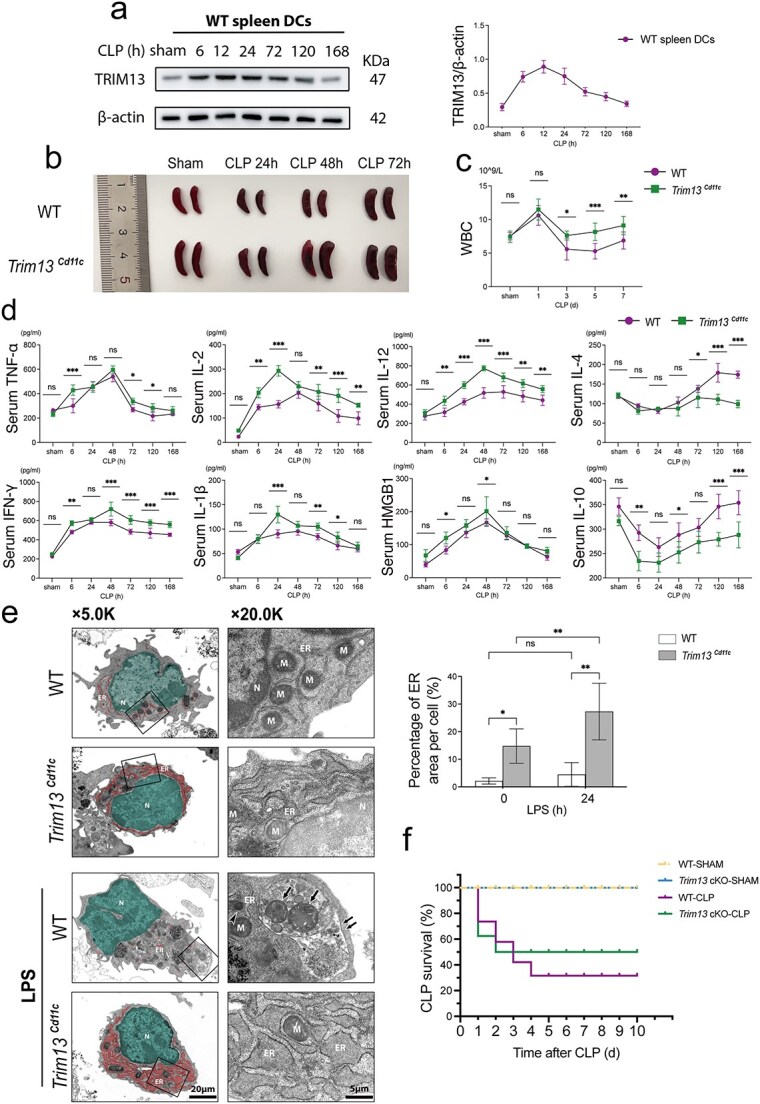
TRIM13 deficiency in splenic DCs alters ER morphology and affects the outcomes of septic mice. (a) Immunoblot analyses revealed TRIM13 expression in primary WT splenic DCs (WT DCs) at the indicated post-CLP time points. The protein levels were normalized to those of β-actin and presented on the right (n = 3). (b) Representative images of whole spleens resected at the indicated post-CLP time points. (c) WBC counts in WT and Trim13 cKO mice at the indicated post-CLP time points (n = 5 mice per group). (d) Serum cytokine levels at the indicated post-CLP time points were quantified by ELISA (n = 5 mice per group). (e) TEM images showing ultrastructural changes in the ER morphology of WT and Trim13Cd11c DCs after stimulation with LPS (1 μg/ml) for 24 h. ER regions are highlighted in red for enhanced visualization, whereas nuclei are stained green. The quantification of the ER areas (n = 3) is shown on the right. AL, autolysosome; AP, autophagosome; EW, endoplasmic reticulum whorl; M, mitochondria; N, nucleus; scale bars: 20 μm and 5 μm. (f) Survival curves following CLP (n = 20 mice per group) demonstrating the impact of TRIM13-deficient DCs on survival outcomes. Data are expressed as means ± SD. ns = not significant; ^*^P < 0.05; ^*^^*^P < 0.01; ^*^^*^^*^P < 0.001. *DCs* dendritic cells, *ER* endoplasmic reticulum, *SD* standard deviation, *WT* wild-type, *TRIM13* tripartite motif 13, *CLP* cecal ligation and puncture, *WBC* white blood cell counts, *ELISA* enzyme-linked immunosorbent assay, *TEM* transmission electron microscopy

To investigate the role of TRIM13 in DC-mediated immunity during sepsis, we first examined its expression in splenic DCs from WT CLP mice. Immunoblot analysis showed a rapid induction of TRIM13, with levels peaking at 12 h post-CLP and subsequently declining by day 5 (Figure 1a).

The deletion of TRIM13 in splenic DCs from *Trim13* cKO mice (*Trim13^Cd11c^* DCs) was confirmed *via* immunoblot analysis (Figure S1a, see online supplementary material). Following CLP, *Trim13* cKO mice displayed significant splenomegaly compared with WT controls (Figure 1b). Consistently, WBC in *Trim13* cKO mice recovered more rapidly from the initial decline (Figure 1c). TRIM13 deficiency in DCs led to an exacerbation of host inflammatory response after CLP, as serum levels of pro-inflammatory cytokines [interleukin (IL)-1β, IL-12, tumor necrosis factor (TNF)-α, and interferon [IFN]-γ] were markedly elevated and most of them persisted for up to 168 h post-CLP. In contrast, WT mice exhibited a pronounced rise in anti-inflammatory cytokines (IL-4 and IL-10) from day 3 after CLP, whereas this compensatory anti-inflammatory response was largely absent in *Trim13* cKO mice (Figure 1d).

Under physiological conditions, the most prominent alteration of *Trim13^Cd11c^* DCs was a substantial increase in the ER abundance with consistent luminal spacing. Following LPS stimulation, both WT and *Trim13^Cd11c^* DCs presented increased cell sizes. In WT DCs, LPS stimulation led to an increase in mitochondrial number and canonical (bulk) autophagy, with slight changes in ER morphology. Conversely, LPS-stimulated *Trim13^Cd11c^* DCs displayed an irregular expansion of ER sheet luminal spacing with an absence of autophagy features (Figure 1e).

To evaluate the impact of TRIM13 deletion in DCs on sepsis outcomes, we compared the mortality rates of WT and *Trim13* cKO mice subjected to CLP. During the first 48 h post-CLP, the mortality of mice in the *Trim13* cKO group was higher than in the WT group. However, the survival of *Trim13* cKO mice was significantly improved than that of WT mice in the following days. A 10-day survival analysis revealed a greater overall survival rate in *Trim13* cKO mice than in WT mice (Figure 1f).

### Trim13 deficiency in DCs promotes the improvement of organ function after early septic insult

**Figure 2:**
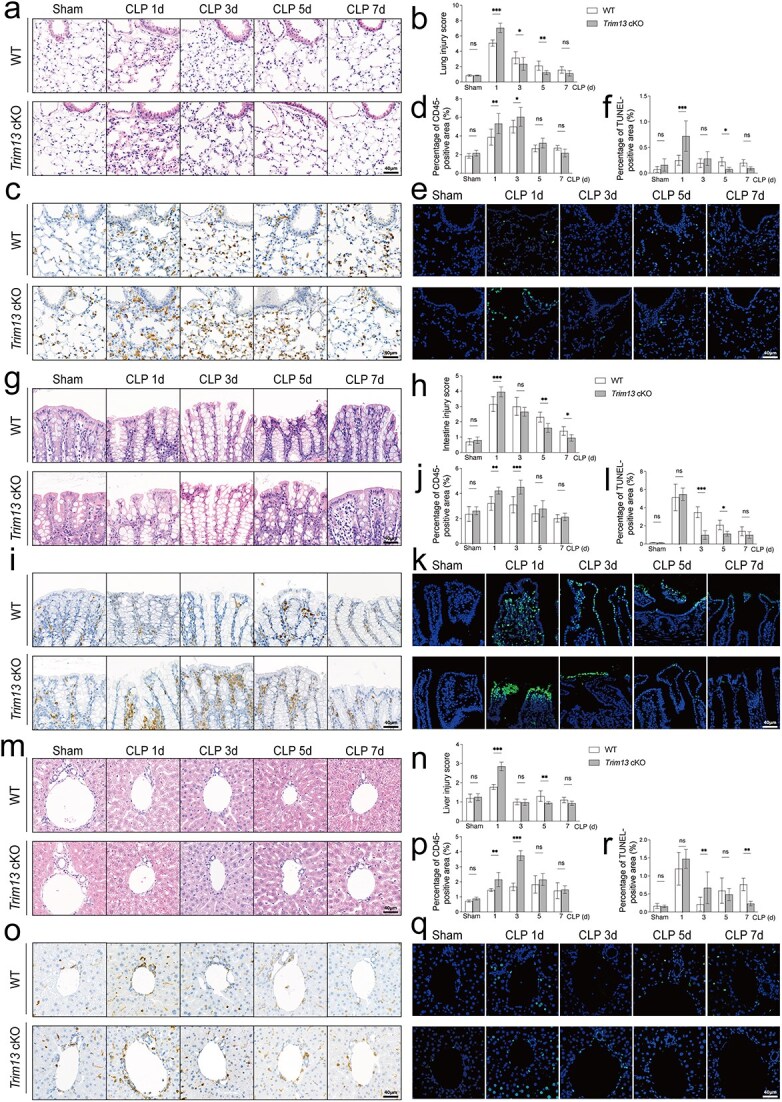
Time-course analysis of organ lesions in WT and *Trim13* cKO CLP mice. Lung (a-f): (a) representative H&E-stained sections of lung tissues, with injury scores quantified in (b). (c) IHC staining of CD45^+^ leukocytes in lung sections, with quantification of leukocyte foci per slide in (d). (e) IF/TUNEL staining of lung sections to detect apoptotic cells, with quantification of apoptotic foci per slide in (f). Intestine (g–l): (g) representative H&E-stained sections of intestinal tissues, with injury scores quantified in (h). (i) IHC staining of CD45^+^ leukocytes in intestinal sections, with quantification of leukocyte foci per slide in (j). (k) IF/TUNEL staining of intestinal sections to detect apoptotic cells, with quantification of apoptotic foci per slide in (l). Liver (m–r): (m) representative H&E-stained sections of liver tissues, with injury scores quantified in (n). (o) IHC staining of CD45^+^ leukocytes in liver sections, with quantification of leukocyte foci per slide in(p). (q) IF/TUNEL staining of liver sections to detect apoptotic cells, with quantification of apoptotic foci per slide in (r). Data are expressed as means ±SD, n = 6. ns = not significant; scale bars: 40 μm. ^*^P < 0.05; ^*^^*^P < 0.01; ^*^^*^^*^P < 0.001. *IF/TUNEL* immunofluorescence with terminal deoxynucleotidyl transferase dUTP nick-end labeling, *IHC* immunohistochemistry, *SD* standard deviation, *WT* wild-type, *CLP* cecal ligation and puncture, *H&E* hematoxylin and eosin

Next, we evaluated organ damage in *Trim13* cKO CLP mice, focusing on the lung, intestine, and liver, with WT mice as controls. At 24 h post-CLP, *Trim13* cKO mice exhibited more severe pulmonary injury than WT mice, characterized by alveolar congestion, hemorrhage, thickened alveolar walls, and a marked increase in apoptotic and infiltrating immune cells (Figure 2a–f). Meanwhile, hematoxylin and eosin (H&E) staining and IF/TUNEL staining revealed exacerbated intestinal injury in *Trim13* cKO mice from 24 to 72 h post-CLP, with IHC staining showing enhanced leukocyte infiltration in intestinal tissues (Figure 2g–l). Similarly, *Trim13* cKO mice exhibited more severe liver injury at 24 h post-CLP. By 72 h post-CLP, the liver damage scores of the *Trim13* cKO mice decreased (Figure 2m–r). Notably, *Trim13* cKO mice displayed more evidence of tissue recovery at later stage, with substantial histological improvement of the lung, intestine, and liver by 168 h post-CLP.

To further evaluate the impact of DC TRIM13 deficiency on organ function, we performed biochemical and physiological assessments of the forementioned organs. Pulmonary edema was quantified by calculating the lung wet-to-dry (W/D) weight ratio. On the first day post-CLP, *Trim13* cKO mice exhibited a significantly higher W/D ratio than WT controls, indicating more severe lung injury. However, this ratio decreased more rapidly in *Trim13* cKO mice from days 5 to 7 (Figure S1b, see online supplementary material). For intestinal barrier integrity, serum diaminoxidase (DAO) and D-lactate (D-LA) levels were measured. DAO levels peaked on day 1 after CLP and were markedly elevated in *Trim13* cKO mice compared with WT mice, consistent with aggravated early intestinal damage. On day 7, DAO and D-LA levels declined significantly in both groups, with no substantial differences between the two groups (Figure S1c and d, see online supplementary material). Hepatic function was assessed by serum alanine aminotransferase (ALT), aspartate aminotransferase (AST), and total bilirubin (TBIL). In line with histological changes, *Trim13* cKO mice displayed higher ALT and AST levels on days 1–3 post-CLP. However, from days 5 to 7, ALT, AST, and TBIL levels declined earlier and more prominently in *Trim13* cKO mice than in WT controls (Figure S1e–g). Coagulation and renal function were also evaluated. The recovery of platelet counts was faster in *Trim13* cKO mice on day 3 post-CLP (Figure S1h, see online supplementary material). Additionally, *Trim13* cKO mice showed significantly lower serum creatinine (Cr) levels, particularly on day 7 post-CLP (Figure S1i, see online supplementary material), indicating improved kidney function.

### Trim13–deficient DCs exhibit a pro-inflammatory phenotype and drive enhanced adaptive immune response during sepsis

**Figure 3:**
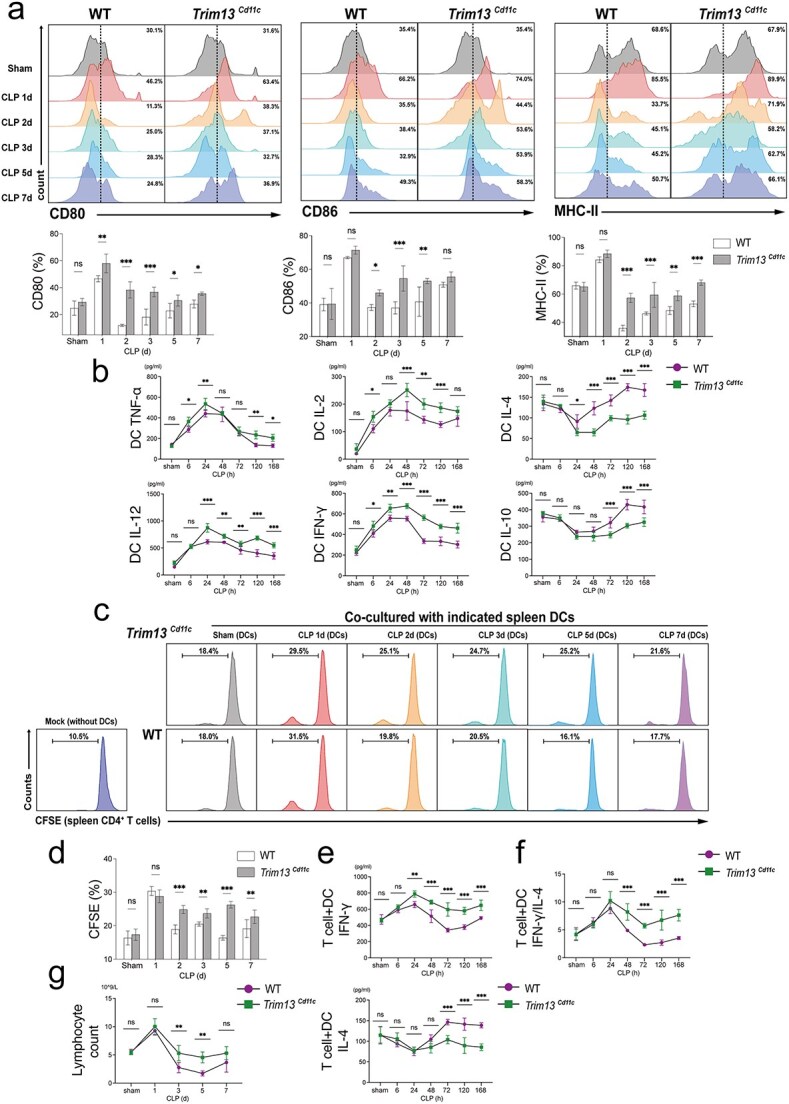
TRIM13 deficiency alters DC activation and T cell immunity during sepsis. (a–f) Splenic DCs isolated from WT and *Trim13* cKO mice at indicated time points following CLP were subjected to subsequent assays. (a) The expression levels of costimulatory molecules, including CD80, CD86, and MHC II, were quantified by flow cytometry (*n* = 3 mice per group). The results are presented below. (b) Cytokine concentrations in DC culture supernatants were quantified by ELISA (*n *= 5 mice per group). (c-d) 5,6-CFSE labeled CD4^+^ T cells co-cultured with the indicated DCs. T cell proliferation was analyzed by flow cytometry (*n* = 3 mice per group). (e) Cytokine concentrations in culture supernatants from CD4^+^ T cells co-cultured with the indicated DCs, quantified by ELISA (*n* = 5 mice per group). (f) IFN-γ/ IL-4 ratio in CD4^+^ T cell-DC co-culture supernatants at the indicated time points (*n* = 5 mice per group). (g) Peripheral blood lymphocyte counts in WT and *Trim13* cKO mice at the indicated post-CLP time points (n = 5 mice per group). Data are expressed as means ± SD. ns = not significant; ^*^P < 0.05; ^*^^*^P < 0.01; ^*^^*^^*^P < 0.001. *DCs* dendritic cells, *SD* standard deviation, *WT* wild-type, *TRIM13* tripartite motif 13, *CLP* cecal ligation and puncture, *ELISA* enzyme-linked immunosorbent assay, *IFN-γ* interferon-*γ*, *IL* interleukin

As professional APCs, MHC II, CD80, and CD86 are crucial for DC antigen presentation and CD4^+^ T cell activation. To investigate the role of TRIM13 in DC functionality during sepsis, splenic DCs were isolated from WT and *Trim13* cKO mice at different time points following CLP. Flow cytometry showed an elevated expression of MHC II, CD80, and CD86 in WT DCs shortly after CLP, followed by a sharp decrease and gradual recovery over time. In contrast, *Trim13^Cd11c^* DCs presented increased CD80, CD86, and MHC II expressions across all post-CLP time points, maintaining relatively stable expression up to 168 h after CLP (Figure 3a).

DCs can shape immune responses by producing cytokines upon encountering pathogens. An ELISA was performed to measure cytokine levels in the culture supernatants of WT and *Trim13^Cd11c^* DCs from CLP mice. Compared with WT DCs, *Trim13^Cd11c^* DCs presented higher levels of pro-inflammatory cytokines, with these levels remaining elevated during late sepsis. Conversely, the levels of anti-inflammatory cytokines (IL-4 and IL-10) were robustly suppressed from 48 to 168 h post-CLP (Figure 3b).

Next, we evaluated the effect of *Trim13^Cd11c^* DCs in mediating T cell proliferation and differentiation. Normal splenic CD4^+^ T cells were labeled with 5,6-carboxyfluorescein succinimidyl ester (CFSE) and cocultured with DCs from WT or *Trim13* cKO CLP mice. Both WT and *Trim13^Cd11c^* DCs effectively induced T cell proliferation at 24 h post-CLP. However, WT DCs rapidly lost this capacity after 24 h, consistent with the observed decrease in costimulatory molecule expression. In contrast, *Trim13^Cd11c^* DCs sustained the ability to activate and promote T cell proliferation throughout the observation period, up to 168 h post-CLP (Figure 3c and d).

Following activation and clonal expansion, DCs play a crucial role in directing effector T cell differentiation, thereby shaping the magnitude and specificity of the immune response. To assess the impact of TRIM13-deficient DCs on T helper (Th) cell polarization, we measured the levels of IFN-γ and IL-4 (signature cytokines produced by Th1 and Th2 cells, respectively) in T cell-DC co-cultured supernatants. T cells cocultured with *Trim13^Cd11c^* DCs exhibited a cytokine profile skewed away from IL-4 and toward IFN-γ production, showing a preferential induction of Th1-type response (Figure 3e and f). In line with these *in vitro* findings, peripheral lymphocyte counts revealed that *Trim13* cKO mice recovered more rapidly from the lymphopenia observed on day 3 post-CLP, returning to baseline earlier than WT counterparts (Figure 3g).

### Trim13 regulates STING signaling in DCs during sepsis

**Figure 4:**
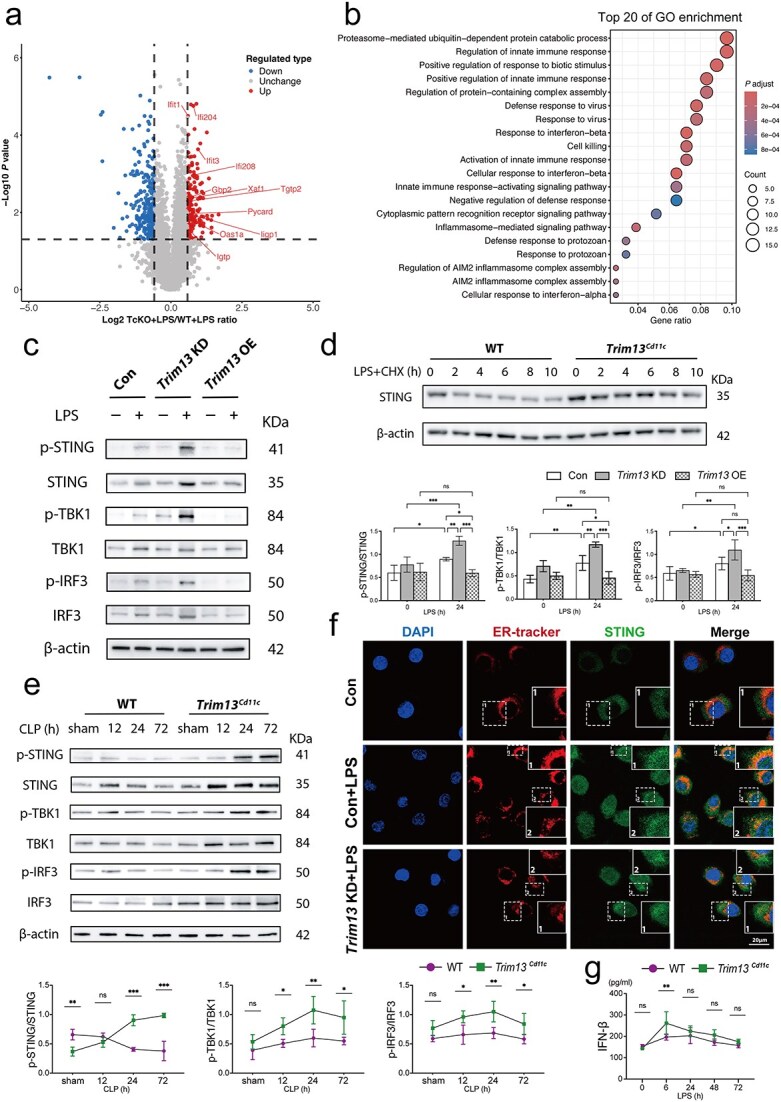
TRIM13 regulates STING signaling in DCs during sepsis. (a) Volcano plots showing differentially expressed proteins in splenic DCs from *Trim13* cKO + LPS group *vs* WT + LPS group. (b) GO analysis highlighting the upregulated biological processes in splenic DCs from *Trim13* cKO + LPS group *vs* WT + LPS group. (c) Immunoblot analysis of DC 2.4 cells with *Trim13* knockdown (*Trim13* KD) or *Trim13* overexpression (*Trim13* OE), demonstrated the impact of *Trim13* knockdown/overexpression on STING signaling. The quantitative results are shown on the right (*n* = 3). (d) Immunoblot analysis of WT and *Trim13*^*Cd11c*^ DCs treated with LPS (1 μg/ml) and CHX (50 μg/ml) for the indicated times revealed STING protein degradation (n = 3). (e) Immunoblot analysis of WT and *Trim13*^*Cd11c*^ DCs at the indicated time points post-CLP revealed protein expression related to STING signaling. The quantitative results are displayed below (n = 3). (f) Representative confocal microscopy images showing the colocalization of ER-tracker (red) and STING (green) in control (con) and *Trim13* KD DC2.4 cells following stimulation with LPS (1 μg/ml, 24 h). Scale bar: 20 μm. (g) IFN-β concentrations in culture supernatants from WT and *Trim13*^*Cd11c*^ DCs were quantified by ELISA following LPS stimulation for the indicated durations (*n* = 5 mice per group). Data are expressed as means ± SD. ns = not significant; ^*^P < 0.05; ^*^^*^P < 0.01; ^*^^*^^*^P < 0.001. *TRIM13* tripartite motif 13, *STING* stimulator of interferon genes, *GO* gene ontology, *LPS* lipopolysaccharide, *DCs* dendritic cells, *SD* standard deviation, *WT* wild-type, *IFN-β* interferon-β, *ELISA* enzyme-linked immunosorbent assay

To elucidate the mechanisms underlying the enhanced functionality of *Trim13^Cd11c^* DCs during sepsis, we conducted comparative proteomic profiling of *Trim13^Cd11c^* and WT DCs following LPS treatment. The biological process enrichment analysis revealed clusters of differentially expressed proteins, with a notable upregulation of mediators involved in the type I IFN response in LPS-treated *Trim13^Cd11c^* DCs (Figure 4a and b). These data suggest that type I IFN-related STING signaling may be potentiated in *Trim13^Cd11c^* DCs upon bacterial challenge [23].

The STING signaling initiates innate immunity by inducing the translocation of STING from the ER to the Golgi, where it activates TANK binding kinase 1 (TBK1) to phosphorylate interferon regulatory factor 3 (IRF3). The nuclear translocation of phosphorylated IRF3 (p-IRF3) promotes type I IFN transcription. To investigate the effect of TRIM13 on STING signaling, DC2.4 cells were infected with either *Trim13* shRNA (*Trim13* KD) or a *Trim13* overexpression (*Trim13* OE) lentiviral vector and then stimulated with LPS. TRIM13 silencing significantly enhanced STING activation, whereas *Trim13* overexpression suppressed this response (Figure 4c).

Using CHX to inhibit protein synthesis, we examined the degradation of STING in WT and *Trim13^Cd11c^* DCs. The protein of STING in WT DCs gradually decreased, while in *Trim13^Cd11^* DCs, it remained at a consistently high level till 10 h after stimuli, indicating a role for TRIM13 in promoting STING degradation (Figure 4d). To investigate the dynamics of STING signaling *in vivo*, splenic DCs were isolated at various time points following CLP. TRIM13 deficiency resulted in the early and sustained activation of the STING signaling after CLP (Figure 4e).

As mentioned above, STING translocates to the Golgi following oligomerization. Thus, the exit of STING from the ER indicated its activation. Immunofluorescence staining confirmed that enhanced STING (green) was released from the ER (red) in *Trim13* KD DC2.4 cells, a finding that is consistent with the increase in STING activation in the absence of TRIM13 (Figure 4f). ELISA revealed a significant increase in the secretion of type I IFN, which are key mediators of STING-dependent immune responses in *Trim13^Cd11c^* DCs (Figure 4g).

### Trim13 deficiency augments gasdermin D-dependent pyroptosis in splenic DCs following sepsis

**Figure 5:**
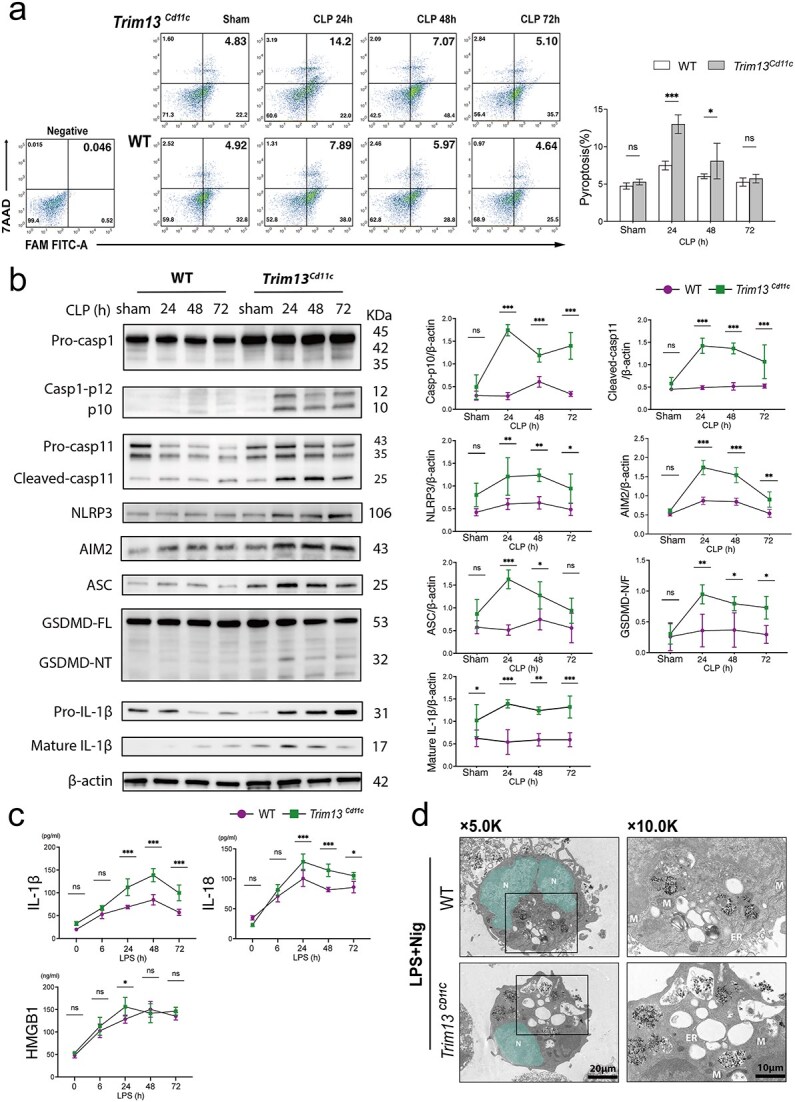
TRIM13 deficiency enhances gasdermin D (GSDMD)-dependent pyroptosis in splenic DCs during sepsis. (a) Time-course flow cytometry analysis of the DC pyroptosis rate following CLP. The splenic DCs were stained for CASP-1 (FAM-FLICA) and 7-AAD. Representative flow cytometry plots are shown on the left, with quantitative data displayed on the right (*n* = 3 mice per group). (b) Immunoblot analysis of WT and *Trim13^Cd11c^* DCs at the indicated time points post-CLP demonstrated that protein expression was related to GSDMD-dependent pyroptosis. The quantitative results are shown on the right (*n* = 3). (c) Concentrations of pyroptosis-related cytokines in culture supernatants from WT and *Trim13^Cd11c^* DCs were quantified by ELISA following LPS stimulation for the indicated durations (*n* = 3 mice per group). (d) Representative TEM images showing ultrastructural changes in WT and *Trim13^Cd11c^* DCs after stimulation with LPS (1 μg/ml) for 24 h and nigericin (nig) (20 μm) for 30 min. Nuclei are highlighted in green for enhanced visualization. N, nucleus; scale bars: 20 μm and 10 μm. Data are expressed as means ± SD. ns = not significant; ^*^P < 0.05; ^**^P < 0.01; ^***^P < 0.001. *DCs* dendritic cells, *SD* standard deviation, *WT* wild-type, *TRIM13* tripartite motif 13, *CLP* cecal ligation and puncture, *WBC* white blood cell counts, *ELISA* enzyme-linked immunosorbent assay, *TEM* transmission electron microscopy, *LPS* lipopolysaccharide

Our proteomic analysis also revealed significant enrichment and upregulation of inflammasome-mediated signaling in LPS-treated *Trim13^Cd11c^* DCs. These data suggest that, upon LPS stimulation, *Trim13^Cd11c^* DCs may release high levels of IL-1 family cytokines and alarmins, potentially driving DC pyroptosis under such conditions [24]. Herein, the pyroptosis rates of splenic DCs from WT and *Trim13* cKO CLP mice were analyzed using dual-label flow cytometry targeting caspase-1 (FAM-FLICA) and 7-AAD (Figure 5a). The results revealed that *Trim13^Cd11c^* DCs presented a significantly elevated pyroptosis rate at 24 h post-CLP compared with WT DCs. At 48 h post-CLP, the pyroptosis rate of *Trim13^Cd11c^* DCs decreased rapidly and returned to the baseline at 72 h. These findings were further corroborated by Western blot analysis, which revealed the significant activation of the gasdermin D (GSDMD)-dependent pyroptosis pathway in *Trim13^Cd11c^* DCs following CLP. This included the activation of the canonical pathway, involving the NLRP3-ASC-caspase-1 complex, and the noncanonical pathway, characterized by the LPS-mediated activation of caspase-11, along with increased GSDMD expression (Figure 5b).

ELISA quantification of cytokines in the culture supernatant of splenic DCs revealed significantly increased levels of IL-1β, IL-18, and high mobility group box-1 (HMGB1) in *Trim13^Cd11c^* DCs post-CLP (Figure 5c) [25, 26]. In addition, the morphology of WT and *Trim13^Cd11c^* DCs was examined using TEM after stimulation with LPS followed by Nig. Compared with WT DCs, *Trim13^Cd11c^* DCs presented pronounced pyroptotic features, including nuclear condensation, cytoplasmic vacuolization, and organelle loss. These results collectively highlight the critical role of TRIM13 in regulating DC pyroptosis following sepsis (Figure 5d).

### Reversal of Trim13 deficiency-induced DC pyroptosis and activation by STING inhibition

**Figure 6:**
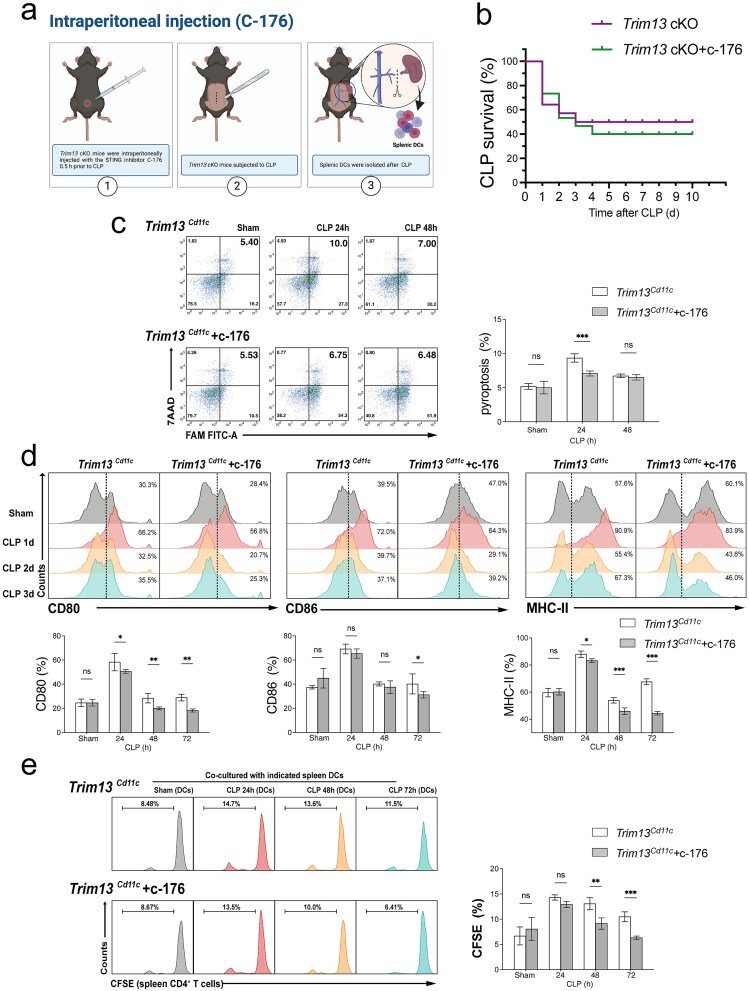
Reversal of TRIM13 deficiency-induced pyroptosis and DC activation by STING inhibition. (a) Scheme for STING inhibitor administration before CLP. (b-e) *Trim13* cKO mice were intraperitoneally injected with the STING inhibitor C-176 (750 nmol in 200 μl of corn oil) 0.5 h prior to CLP. Survival data were collected for analysis. Splenic DCs were isolated at the indicated post-CLP time points for subsequent analyses. (b) Survival curves following CLP (*n* = 20 mice per group), demonstrating the impact of C-176 on the outcomes of WT and *Trim13* cKO mice. (c) Flow cytometry analysis of splenic DC pyroptosis rate at the indicated post-CLP time points (*n* = 3 mice per group). DCs were stained for CASP-1 (FAM-FLICA) and 7-AAD. Representative flow cytometry plots are shown on the left, with quantitative data presented on the right. (d) Flow cytometry analysis of the expression levels of CD80, CD86, and MHC II in splenic DCs (*n* = 3 mice per group). The results are displayed below. (e) the proportion of divided CD4^+^ T cells stained for CFSE was evaluated using flow cytometry (*n* = 3 mice per group). The quantitative results are shown on the right. Data are expressed as means ± SD. ns = not significant; ^*^P < 0.05; ^**^P < 0.01; ^***^P < 0.001. *DCs* dendritic cells, *SD* standard deviation, *WT* wild-type, *TRIM13* tripartite motif 13, *CLP* cecal ligation and puncture

To clarify whether STING serves as a key mediator in TRIM13 deficiency-induced DC activation, we evaluated the functional alterations with DCs following STING inhibition *in vivo*. *Trim13* cKO mice were administered the STING inhibitor C-176 intraperitoneally 0.5 h prior to CLP (Figure 6a). Notably, STING inhibition improved early survival in *Trim13* cKO mice but increased delayed mortality, resulting in a higher overall mortality rate (Figure 6b). Splenic DCs were subsequently isolated at different time points post-CLP for analysis. STING inhibition significantly reduced the pyroptosis rate of DCs at 24 h post-CLP (Figure 6c). Treatment with C-176 attenuated the upregulated expression of costimulatory molecules, including CD80, CD86, and MHC II, after septic challenge (Figure 6d). Moreover, C-176 administration suppressed the increase in T cell proliferation caused by DC TRIM13 deficiency (Figure 6e).

### Trim13-mediated SEL1L-HRD1 ERAD suppressor of lin-12-like-HMG-CoA reductase degradation 1 endoplasmic reticulum-associated degradation and ER-phagy in septic DCs

**Figure 7:**
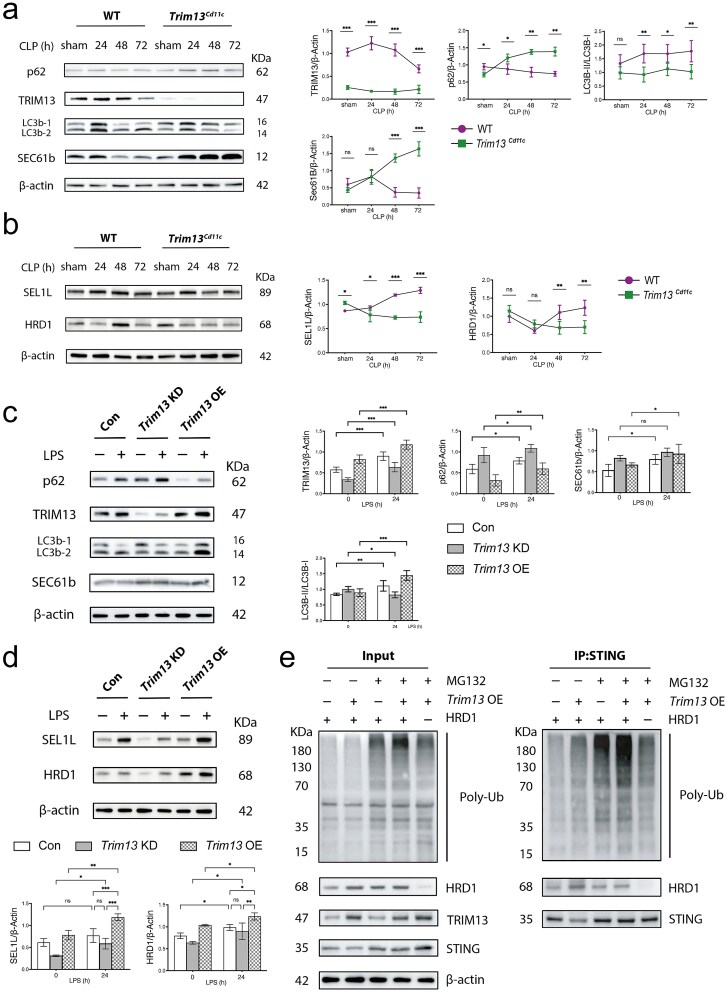
TRIM13 promotes SEL1L-HRD1 endoplasmic reticulum-associated degradation (ERAD) and ER-selective autophagy (ER-phagy) in DCs upon LPS stimulation. (a) Immunoblot analysis of ER-phagy-related protein expression in WT and *Trim13^Cd11c^* DCs at the indicated post-CLP time points (*n* = 3). The quantitative results are shown on the right. (b) Immunoblot analysis of ERAD-related protein expression in WT and *Trim13*^*Cd11c*^ DCs at the indicated post-CLP time points (n = 3) (*n* = 3). The quantitative results are shown on the right. (c) Immunoblot analysis of ER-phagy-related protein expression in * Trim13* KD and *Trim13* OE DC2.4 cells following stimulation with LPS (1 µg/ml, 24 h) (n =3). The quantitative results are shown on the right. (d) Immunoblot analysis of ERAD-related protein expression in *Trim13* KD and *Trim13* OE DC2.4 cells following stimulation with LPS (1 μg/ml, 24 h) (*n* = 3). The quantitative results are shown below. (e) Immunoblot analysis of STING ubiquitination following STING immunoprecipitation in *Trim13* OE DC2.4 cells treated with MG-132 (10 μm, 12 h) or *Hrd1* knockdown. Data are expressed as means ± SD. ns = not significant; ^*^P < 0.05; ^**^P < 0.01; ^***^P < 0.001. *DCs* dendritic cells, *SD* standard deviation, *ER* endoplasmic reticulum, *WT* wild-type, *TRIM13* tripartite motif 13, *CLP* cecal ligation and puncture, *STING* stimulator of interferon genes

To assess TRIM13-mediated ER-phagy and ERAD in DCs, splenic DCs were isolated from WT and *Trim13* cKO mice following CLP. Immunoblot analysis revealed that WT DCs increased ER-phagy and ERAD activity at 24–48 h post-CLP. In contrast, *Trim13^Cd11c^* DCs presented impaired ER-phagy and ERAD during the same period (Figure 7a and b). Under LPS stimulation, *Trim13* OE DC2.4 cells presented increased TRIM13 and LC3B-II/I expression and decreased levels of p62, indicating increased autophagy. Conversely, *Trim13* KD cells showed the opposite trend after LPS treatment (Figure 7c). *Trim13* overexpression increased SEL1L and HRD1 levels, whereas *Trim13* knockdown significantly reduced SEL1L and HRD1 levels following LPS stimulation (Figure 7d).

It has been demonstrated that the SEL1L-HRD1 ERAD is an important mechanism controlling STING ubiquitination and degradation [27]. To investigate whether TRIM13 modulates STING ubiquitination through this complex, we overexpressed TRIM13 and simultaneously silenced HRD1 in DC2.4 cells, followed by MG-132 treatment to stabilize ubiquitinated proteins. STING ubiquitination was then assessed after immunoprecipitation.

*Trim13* overexpression substantially increased STING ubiquitination, particularly in the presence of MG132, whereas *Hrd1* knockdown attenuated this effect, indicating that TRIM13 facilitates STING turnover *via* the SEL1L-HRD1 ERAD machinery and thereby influences its degradation and downstream signaling (Figure 7e).

### Trim13 regulates STING signaling by ERAD and ER-phagy in DCs after sepsis

**Figure 8:**
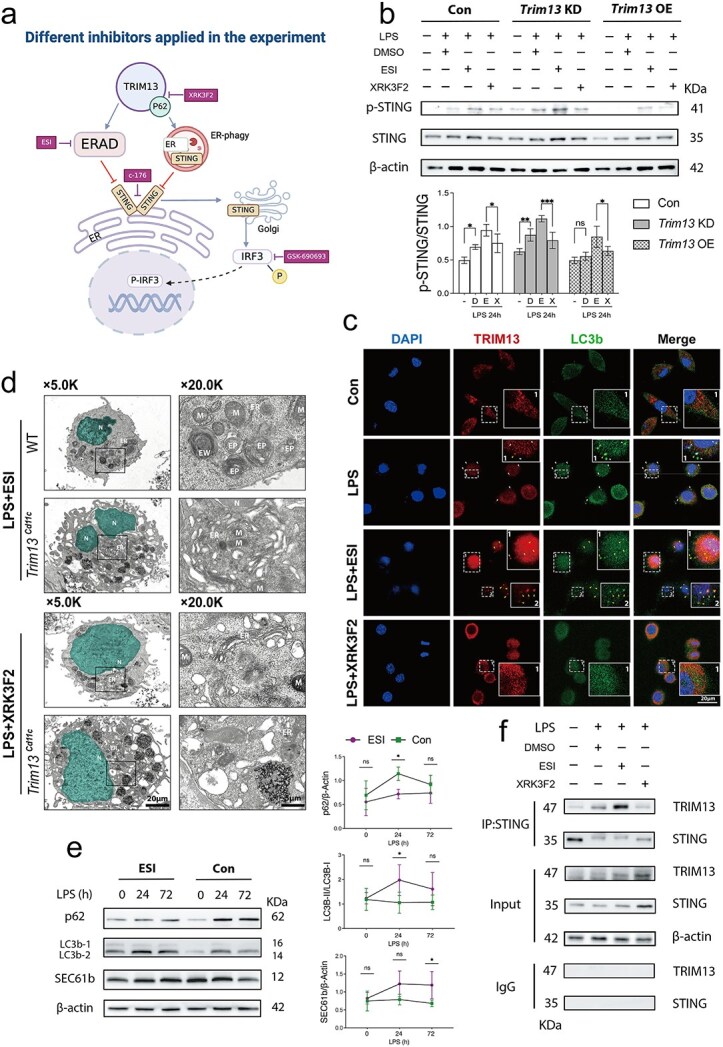
TRIM13 regulates DC STING signaling by ERAD and ER-phagy in sepsis. (a) Different inhibitors were applied in the experiment. (b) Immunoblot analysis showing STING phosphorylation levels in con, *Trim13* KD and *Trim13* OE DC2.4 cells under various stimulation conditions (LPS, 1 μg/ml, 24 h; DMSO, 24 h; ESI, 20 μm, 4 h; XRK3F2, 10 μm, 24 h) (*n* = 3). The quantitative results are displayed below. (c) Representative confocal microscopy images revealed the colocalization of TRIM13 (red) and LC3 (green) in con DC2.4 cells following stimulation with LPS (1 μg/ml, 24 h), ESI (20 μm, 4 h), or XRK3F2 (10 μm, 24 h). Scale bar: 20 μm. (d) TEM images showing ultrastructural alterations in the ER morphology of WT and *Trim13^Cd11c^* DCs after stimulation with LPS (1 μg/ml, 24 h), ESI (10 μm, 4 h), or XRK3F2 (10 μm, 24 h). Nuclei are highlighted in green for enhanced visualization. EP, ER-phagy; scale bars: 20 μm and 5 μm. (e) Immunoblot analysis of con DC2.4 cells stimulated with LPS (1 μg/ml, 24 h) and treated with ESI (20 μm, 4 h) revealed the effect of ERAD inhibition on the expression of ER-phagy-related proteins. The quantitative data are shown on the right. Data are expressed as means ± SD. ns = not significant; ^*^P < 0.05; ^**^P < 0.01. (f) Coimmunoprecipitation (Co-IP) was used to assess the interaction between TRIM13 and STING in con DC 2.4 cells stimulated with LPS (1 μg/ml, 24 h), DMSO, ESI (20 μm, 4 h), or XRK3F2 (10 μm, 24 h). Cell lysates were immunoprecipitated with an anti-STING antibody and immunoblotted with an anti-TRIM13 antibody. IgG controls were included to confirm specificity. *DCs* dendritic cells, *ER* endoplasmic reticulum, *SD* standard deviation, *WT* wild-type, *TRIM13* tripartite motif 13, *TEM* transmission electron microscopy, *STING* stimulator of interferon genes, *ERAD* endoplasmic reticulum-associated degradation, *LPS* lipopolysaccharide

To investigate the involvement of ERAD and ER-phagy in TRIM13-mediated STING regulation, we first assessed STING activation following LPS stimulation. As expected, *Trim13* overexpression suppressed STING signaling activation. However, this response was reversed upon ERAD inhibition and only partially attenuated by ER-phagy inhibition, indicating that ERAD is the primary pathway involved (Figure 8a and b).

Following LPS stimulation, immunofluorescence analysis of DC2.4 cells revealed a significant enhancement in autophagic activity; however, TRIM13-mediated ER-phagy was observed at only a minimal level (Figure 8c). This observation was consistent with the TEM images of primary WT DCs, which lacked evidence of ER-phagy under similar conditions. Interestingly, treatment with the ERAD inhibitor ESI induced the significant colocalization of TRIM13 with LC3 in DC2.4 cells after LPS stimulation. Given that TRIM13 is an ER membrane protein, this colocalization provided evidence of TRIM13-mediated ER-LC3 interactions (Figure 8c). Correspondingly, TEM analysis of primary WT DCs treated with ESI revealed clear ER-phagy features, including ER portions within the autophagosome. In contrast, *Trim13^Cd11c^* DCs exhibited cellular swelling but lacked identifiable ER-phagy features. Next, WT DCs were cotreated with LPS and the p62 (ZZ domain) inhibitor XRK3F2. Under these conditions, both canonical autophagy and ER-phagy were absent in WT DCs (Figure 8d). In addition, immunoblot analysis of ESI-treated DCs revealed an increase in the expression of ER-phagy proteins, further confirming the role of TRIM13 in regulating ER-phagy in the context of septic insult (Figure 8e).

Since the abovementioned findings reveal that TRIM13-induced ER-phagy is enhanced upon ERAD impairment, we further investigated whether ER-phagy compensates for the ability of ERAD to regulate STING degradation. Co-IP experiments confirmed that LPS treatment modestly enhanced the interaction between STING and TRIM13. This interaction was significantly amplified by ERAD inhibition, and the disruption of ER-phagy notably attenuated the binding activity. This finding provides direct evidence that TRIM13 facilitates STING degradation through ER-phagy when ERAD is compromised (Figure 8f).

### Trim13 deficiency-induced STING activation enhances p-IRF3 nuclear translocation and NLRP3 expression in DCs following sepsis

**Figure 9:**
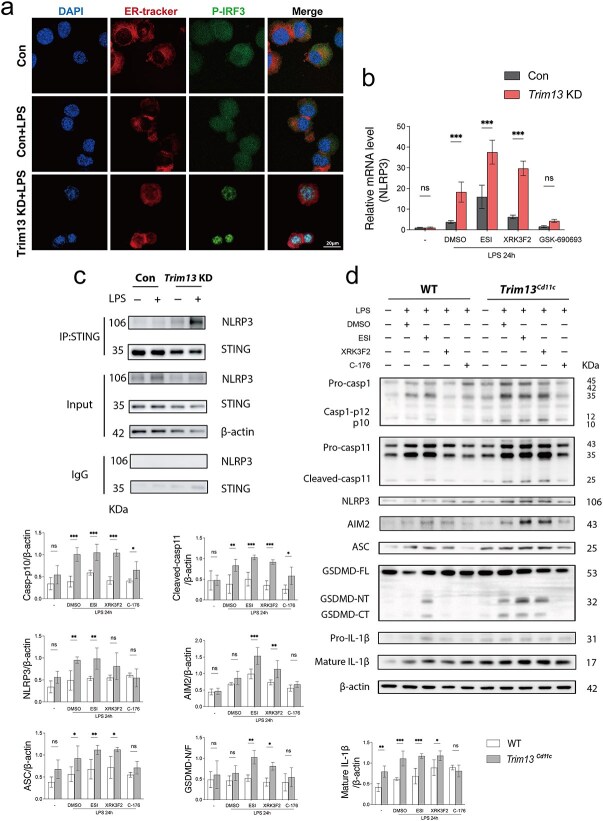
TRIM13 silencing enhances p-IRF3 nuclear translocation and upregulates NLRP3 expression in DCs following sepsis. (a) Representative confocal microscopy images showing the nuclear translocation of p-IRF3 (green) in con and *Trim13* KD DC2.4 cells after LPS stimulation (1 μg/ml, 24 h). DAPI: Blue; ER-tracker: Red; scale bar: 20 μm. (b) qPCR analysis was performed to assess NLRP3 mRNA expression in con and *Trim13* KD DC2.4 cells stimulated with LPS (1 μg/ml, 24 h), DMSO, ESI (10 μm, 4 h), XRK3F2 (10 μm, 24 h) or GSK-690693 (15 μm, 24 h). Data are normalized to GAPDH expression as an internal control and presented as the relative fold change using the ΔΔCt method. Data are expressed as means ± SD. ns = not significant; ^*^P < 0.05; ^**^P < 0.01; ^***^P < 0.001. (c) Co-IP was used to assess the interaction between NLRP3 and STING in con and *Trim13* KD DC2.4 cells stimulated with LPS (1 μg/ml, 24 h). Cell lysates were immunoprecipitated with an anti-STING antibody and probed with an anti-NLRP3 antibody. IgG controls were included to confirm specificity. (d) Immunoblot analysis of WT and *Trim13^Cd11c^* DCs revealed protein expression associated with GSDMD-dependent pyroptosis after various stimulation (LPS, 1 μg/ml, 24 h; DMSO, 24 h; ESI, 10 μm, 4 h; XRK3F2, 10 μm, 24 h; C-176, 10 μm, 1 h pretreatment) (*n* = 3). The quantitative results are displayed on the left and below. *DCs* dendritic cells, *ER* endoplasmic reticulum, *SD* standard deviation, *TRIM13* tripartite motif 13, *STING* stimulator of interferon genes, *LPS* lipopolysaccharide

Previous studies have demonstrated that STING activation facilitates the nuclear translocation of p-IRF3, subsequently increasing NLRP3 expression and resulting in pyroptosis. To assess p-IRF3 translocation, immunofluorescence staining was conducted to analyze its subcellular localization in DC2.4 cells. Following LPS stimulation, the control (Con) DC2.4 cells presented minimal nuclear translocation of p-IRF3; in contrast, *Trim13* KD DC2.4 cells exhibited a robust increase in p-IRF3 nuclear translocation (Figure 9a). QPCR analysis of NLRP3 expression in Con and *Trim13* KD DC2.4 cells provided additional evidence supporting these observations. Compared with their counterparts, *Trim13* KD DC2.4 cells presented significantly increased NLRP3 mRNA expression following LPS stimulation. The pharmacological inhibition of IRF3 phosphorylation with GSK-690693 significantly downregulated the LPS-induced expression of NLRP3 in *Trim13* KD DC2.4 cells (Figure 9b).

STING has been reported to reduce NLRP3 degradation by directly binding to NLRP3, thereby enhancing inflammasome assembly and activation [28]. To investigate the potential interaction, we performed Co-IP assays using DC2.4 cells following LPS stimulation. A pronounced increase in STING-NLRP3 interactions was detected in *Trim13* KD cells compared with control DC2.4 cells (Figure 9c). Next, we isolated splenic DCs from WT and *Trim13* cKO mice and then used ESI, XRK3F2 and C-176 to inhibit ERAD, ER-phagy and STING activation, respectively following LPS treatment. Notably, the inhibition of ERAD significantly augmented DC pyroptosis, consistent with earlier findings that TRIM13 deficiency promoted STING activation primarily through ERAD impairment. However, treatment with the STING inhibitor C-176 effectively reversed the upregulation of pyroptosis-related protein expression in *Trim13^Cd11c^* DCs, highlighting the role of STING activation in driving this process (Figure 9d).

## Discussion

Sepsis is a life-threatening condition of immune dysregulation and organ dysfunction, characterized by both excessive inflammation and profound immunosuppression. Despite tremendous efforts at hyperinflammation control, the consistent failure of anti-inflammatory therapies in clinical trials highlights the importance of restoring immune competence during immunosuppression [29, 30]. This sustained immunosuppression is commonly driven by profound alterations in immune cells, including their depletion, functional anergy, and shift toward an anti-inflammatory phenotype. As key initiators of adaptive immunity, DC dysfunction plays a critical role in this process, and therapeutic strategies that restore DC function hold promise for immune reconstitution and host protection.

Here, we delineate an unrecognized role of TRIM13 restriction in upregulating DC function during sepsis. Upon pathogenic stress, TRIM13 deficiency enhances STING signaling in septic DCs, thereby amplifying DC-mediated antigen presentation, promoting naïve T cell priming and clonal expansion, as well as lymphocyte recovery. These combined effects facilitate tissue repair, improve organ function, and ultimately enhance survival outcomes in septic mice. Mechanistically, in normal conditions, TRIM13 suppresses DC function by inhibiting STING accumulation and signaling, primarily through the degradative SEL1L-HRD1 ERAD.

Consistent with previous findings that upregulated TRIM13 limits STING and type I interferon signaling during viral infection [13, 31–34], we found that TRIM13 expression is also upregulated in DCs following CLP-induced sepsis. To test its *in vivo* significance, we generated DC-specific *Trim13* knockout (*Trim13* cKO) mice and subjected both WT and *Trim13* cKO mice to CLP [35]. Compared with *Trim13* cKO mice, WT mice subjected to severe CLP (over 60% mortality on day 10) in our study exhibited features of immune paralysis on day 3 post-CLP, including elevated serum anti-inflammatory cytokines, lymphopenia, reduced MHC-II expression on DCs, and impaired T cell proliferation. Meanwhile, survival analysis revealed distinct survival patterns between WT and *Trim13* cKO mice. *Trim13* cKO mice exhibited transiently increased mortality in the early hyperinflammatory phase, but showed improved survival after day 3, resulting in a lower overall mortality rate. Serum cytokine profiling further corroborated these findings, as *Trim13* cKO mice maintained elevated levels of proinflammatory cytokines and reduced anti-inflammatory mediators during late-phase immunosuppression.

Beyond survival outcomes, TRIM13 deficiency reshaped DC morphology and function. TEM images specifically revealed an expansion of ER sheets in *Trim13^Cd11c^* DCs. This morphological alteration reveals an accumulation of ER proteins and a reduction in ER turnover [33, 36, 37]. Functionally, *Trim13^Cd11c^* DCs maintained high expression of CD80, CD86, and MHC-II, together with persistent proinflammatory cytokine secretion. This phenotype endowed *Trim13^Cd11c^* DCs with a greater capacity to promote naïve T cell activation and clonal expansion. An increased IFN-γ/IL-4 ratio further indicated a shift toward Th1 polarization in effector cell differentiation. Importantly, while WT mice developed an immunosuppressive state on day 3 post-CLP, DC activation and cytokine production remained robust in *Trim13* cKO mice, coinciding with a rapid recovery of peripheral lymphocyte counts.

In addition to immune modulation, TRIM13-deficient DCs conferred benefit on the improvement of organ function. Histological, IHC, and IF/TUNEL staining analyses revealed improved tissue structural integrity in *Trim13* cKO mice on day 7 post-CLP, paralleled by recovery of coagulation, hepatic, and renal parameters. These findings align with previous reports that the restoration of immune response through anti-PD-1 or anti-PD-L1 antibody administration improves organ function and survival in septic mice [6, 38–40].

Given that infectious insults induce DC death in the pathogenesis of sepsis [41–43]. We analyzed our proteomic data in this study, which revealed an increased susceptibility of *Trim13^Cd11c^* DCs to pyroptosis under such conditions. Notably, the pyroptotic stress was transient and did not substantially reduce the total number of DCs. Meanwhile, pyroptosis is highly proinflammatory; the inflammatory mediators released from pyroptotic cells amplify DC maturation and immune activation, as demonstrated in DC-pyroptosis-based antitumor immunity [44, 45]. However, this transient overactivation also magnified early tissue injury and poor outcome, implicating the importance of intervention timing if TRIM13 is targeted.

To define the downstream signaling, our proteomic and immunoblotting data revealed that the STING pathway was among the most significantly altered in *Trim13^Cd11c^* DCs during sepsis. This finding aligns with reports that TRIM13 negatively regulates STING and type I IFN signaling [13, 34]. STING signaling not only shapes immune activation but also regulates cell death [46]. We therefore hypothesized that enhanced STING signaling may drive the pyroptosis in *Trim13^Cd11c^* DC. Emerging evidence has elucidated several mechanisms through which STING mediates pyroptosis. Primarily, STING promotes NLRP3 transcription *via* increased p-IRF3 nuclear translocation. Concurrently, STING facilitates inflammasome activation by directly binding with NLRP3, stabilizing the inflammasome complex and preventing its degradation [28, 46, 47]. In this context, our results revealed that in DCs, TRIM13 deficiency enhanced ER STING activation and nuclear translocation of p-IRF3 following LPS challenge, increased NLRP3 transcription, and strengthened the STING-NLRP3 interaction, effects that were reversed by the p-IRF3 inhibitor GSK-690693. Moreover, the STING inhibitor C-176 and ERAD inhibitor ESI reduced STING activation and significantly decreased GSDMD expression in *Trim13^Cd11c^* DCs, respectively. These findings confirm that TRIM13 can modulate DC pyroptosis through the STING-NLRP3-GSDMD axis in the context of sepsis.

*In vivo,* administering the STING inhibitor C-176 to CLP mice reduced early DC pyroptosis, attenuated sustained DC activation at the late stage, and increased mortality in *Trim13* cKO CLP mice, indicating that STING pathway activation is involved in TRIM13-induced DC pyroptosis and functional alterations in septic mice.

Finally, we investigated how TRIM13 orchestrated ER quality control to regulate STING degradation. Overexpression of *Trim13* in DCs promoted STING ubiquitination and degradation, an effect that could be reversed by ERAD inhibition, but slightly mitigated by ER-phagy inhibitor, indicating a direct role of TRIM13 in facilitating STING degradation through ERAD. Mechanistically, TRIM13 enhances STING ubiquitination *via* the SEL1L-HRD1 complex, thereby promoting its proteasomal degradation and attenuating downstream signaling. These findings align with previous reports identifying SEL1L-HRD1 ERAD as a crucial suppressor of STING activation under both basal and inflammatory conditions [27].

While TRIM13-mediated ERAD appears to be the dominant pathway for STING degradation, our findings suggest that ER-phagy may act as a complementary mechanism under conditions of ERAD impairment. Typically, TRIM13-induced ER-phagy is dependent on the formation of oligomeric p62-TRIM13 complexes, which are initiated by the binding of p62 *via* its ZZ domain to the N-terminal L-arginine (Nt-Arg) residue [48]. A previous report attempted to investigate the involvement of ER-phagy in TRIM13-mediated STING degradation on the basis of the complete knockout of p62; however, a definitive conclusion has yet to be reached [13]. Given that p62 is essential for both canonical autophagy and selective autophagy, it remains unclear whether the autophagic degradation of STING is specifically attributable to TRIM13. In this context, we employed the p62 ZZ domain inhibitor XRK3F2, minimizing the impact on other autophagic processes [49–51]. However, treatment with XRK3F2 failed to increase STING activation in cells following LPS stimulation and slightly mitigated the reduction in STING accumulation caused by *Trim13* overexpression. Interestingly, when ERAD inhibitors were applied, the ER-phagy-related protein expression and TRIM13-STING interaction were markedly upregulated. This finding was further supported by IF and TEM imaging, which revealed clear ER-phagy morphological features in splenic DCs under the same conditions. These results are in accordance with a recent report that the ERAD inhibition redirects ERAD clients to degradation *via* FAM134B-mediated ER-phagy [52].

While this study provides valuable insights into the role of TRIM13 in regulating DC activation and pyroptosis during sepsis, several limitations should be considered. First, in septic animals, DC pyroptosis predominantly occurs during the early phase of infection. The mechanisms underlying this temporal context remain unclear and warrant further investigation. Second, pyroptosis represents only one mode of DC cell death. The decline in DC counts observed in CLP *Trim13* cKO mice may involves multiple cell death pathways. Therefore, future studies should explore the role of TRIM13 in regulating diverse DC death mechanisms to gain a more comprehensive understanding of its impact on immune homeostasis during sepsis. Third, the DC-specific *Trim13* cKO mice used in this study exhibited significantly reduced fertility and a high proportion of female offspring. If feasible, generating a DC-specific *Trim13* and *Sting* double-knockout mice could further validate the findings. Fourth, while our findings using the CLP model demonstrate the potential role of TRIM13 in modulating DC response during Gram-negative sepsis, validation in alternative models (e.g. Gram-positive bacteria, fungi, or viruses) is warranted to extend these insights to diverse pathogen contexts. Finally, as this study was primarily conducted in animal models, its findings require further validation in clinically relevant models to ensure therapeutic potential in human sepsis.

## Conclusions

In summary, this study identified TRIM13 as a negative regulator of persistent DC activation, which dampens the host inflammatory response during sepsis. TRIM13 upregulates rapidly in DCs post-infection, limiting STING signaling through SEL1L-HRD1 EARD-mediated ubiquitination and degradation. Impaired ERAD triggers TRIM13-induced ER-phagy as compensation. Conversely, TRIM13 deletion amplifies DC STING-IRF3 signaling, enhancing DC function but inducing transient pyroptosis that worsens acute inflammation and tissue injury. In the long term, TRIM13 deficiency enhances effector T cell activation and proliferation, increases pro-inflammatory cytokine production and lymphocyte counts, thereby improving organ function and ultimately promoting overall survival.

## Abbreviations

ASC, apoptosis-associated speck-like protein containing a CARD; ER, endoplasmic reticulum; ERQC, ER quality control; ERAD, ER-associated degradation; ER-phagy, ER-selective autophagy; GSDMD, gasdermin D; HMGB1, high mobility group box-1; IRF3, interferon regulatory factor 3; ISGs, interferon-stimulated Genes; LPS, lipopolysaccharide; MHC, major histocompatibility complex; Nig, nigericin; NLRP3, NLR family pyrin domain containing 3; SEL1L-HRD1, suppressor of lin-12-like (SEL1L)HMG-CoA reductase degradation 1 (HRD1); STING, stimulator of interferon genes; TBK1, TANK binding kinase 1; TRIM13, tripartite motif 13.

## Supplementary Material

Figure_S1_tkaf077
