# Supplementary material for: Tripartite motif 13 orchestrates endoplasmic reticulum-associated degradation and endoplasmic reticulum-phagy to modulate dendritic cell-mediated immune responses in sepsis
Source: Burns Trauma. 2025 Dec 8;14:tkaf077. doi: 10.1093/burnst/tkaf077 (PMC12870119; doi:10.1093/burnst/tkaf077)
Supplement: Figure_S1_tkaf077 [file figure_s1_tkaf077.pdf]

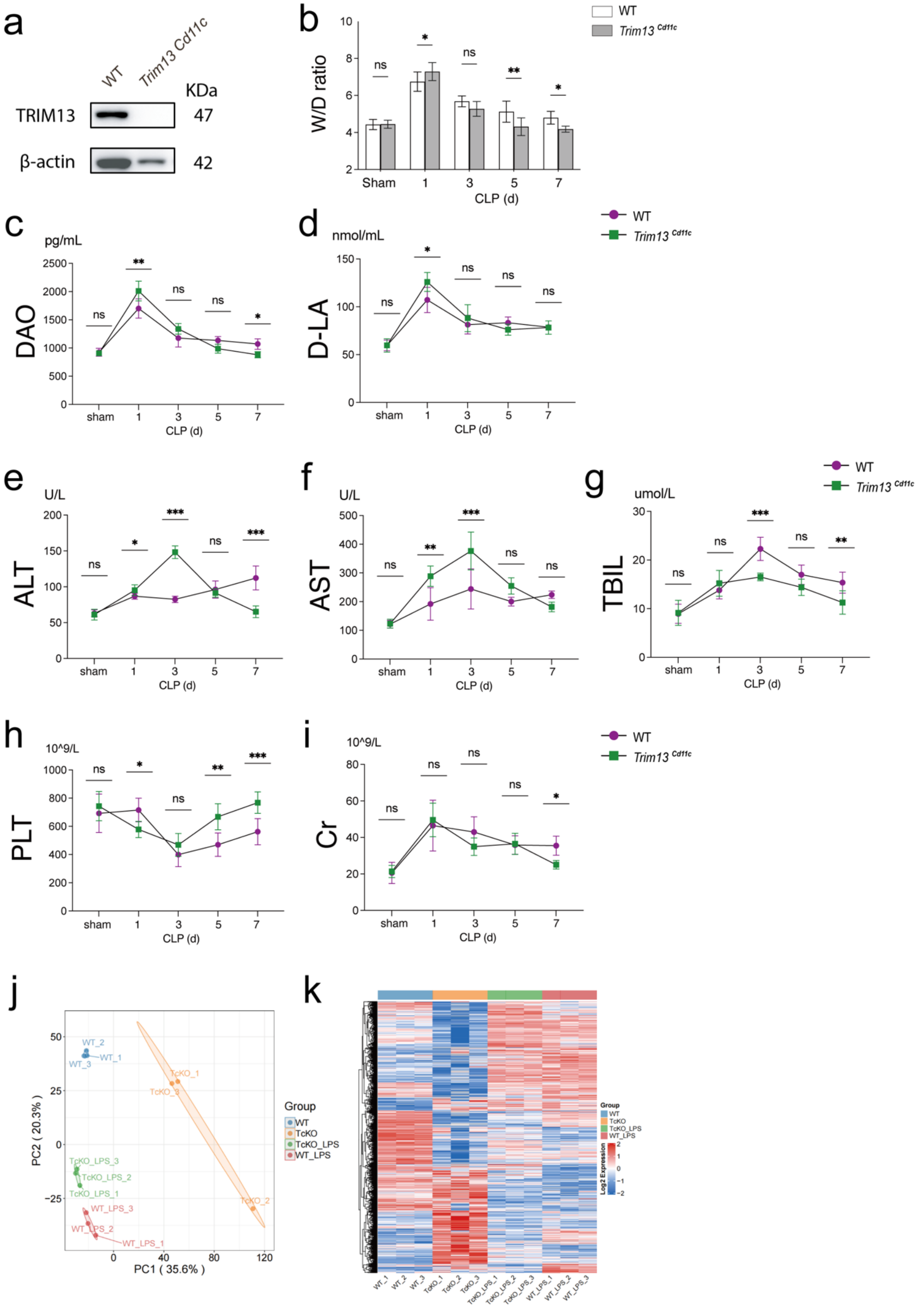

Supplementary Figure 1. Effect of TRIM13 knockout and physiological parameters in WT

and *Trim13* cKO mice (a) Immunoblotting of primary *Trim13*^{Cd11c} splenic DCs revealed the effect of TRIM13 knockout. (b) Quantification of the lung wet-to-dry ratio (W/D ratio) in WT and *Trim13* cKO mice at the indicated post-CLP time points (n = 5 mice per group). (c) Quantification of the serum diamine oxidase (DAO) in WT and *Trim13* cKO mice at the indicated post-CLP time points (n = 5 mice per group). (d) Quantification of the serum D-lactic acid (D-LA) in WT and *Trim13* cKO mice at the indicated post-CLP time points (n = 5 mice per group). (e) Quantification of the serum alanine aminotransferase (ALT) in WT and *Trim13* cKO mice at the indicated post-CLP time points (n = 5 mice per group). (f) Quantification of the serum aspartate aminotransferase (AST) in WT and *Trim13* cKO mice at the indicated post-CLP time points (n = 5 mice per group). (g) Quantification of the serum total bilirubin (TBIL) in WT and *Trim13* cKO mice at the indicated post-CLP time points (n = 5 mice per group). (h) Quantification of platelet counts (PLT) in WT and *Trim13* cKO mice at the indicated post-CLP time points (n = 5 mice per group). (i) Quantification of creatinine (Cr) in WT and *Trim13* cKO mice at the indicated post-CLP time points (n = 5 mice per group). (j) Principal component analysis (PCA) of protein expression profiles comparing WT (WT DCs) + LPS and TcKO (*Trim13*^{Cd11c} DCs) + LPS groups. (k) Heatmap visualization of differentially expressed proteins between WT + LPS and TcKO + LPS groups. Data are presented as mean ± SD. ns = not significant; **P*<0.05; ***P*<0.01; ****P*<0.001.
